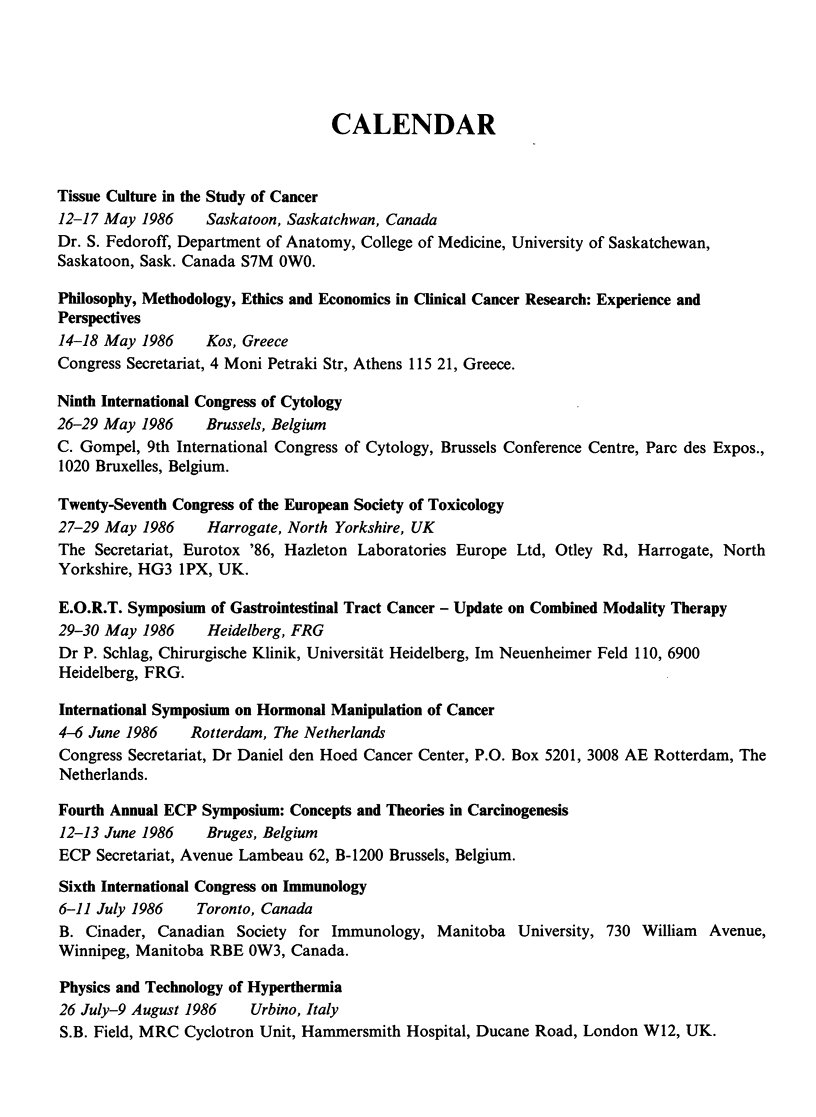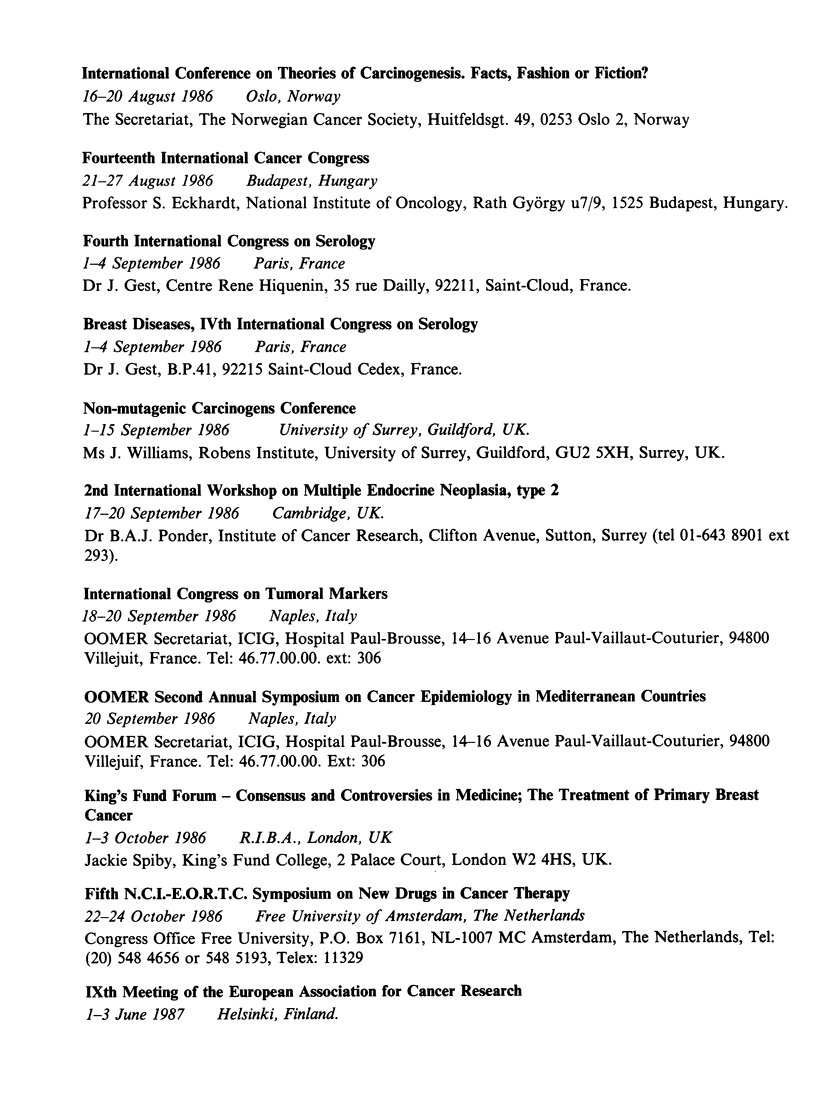# Calendar

**Published:** 1986-05

**Authors:** 


### CALENDAR

**Tissue Culture in the Study of Cancer**
*12–17 May 1986 Saskatoon, Saskatchwan, Canada*
Dr. S. Fedoroff, Department of Anatomy, College of Medicine, University of Saskatchewan, Saskatoon, Sask. Canada S7M 0W0.

**Philosophy, Methodology, Ethics and Economics in Clinical Cancer Research: Experience and Perspectives**
*14–18 May 1986 Kos, Greece*
Congress Secretariat, 4 Moni Petraki Str, Athens 115 21, Greece.

**Ninth International Congress of Cytology**
*26–29 May 1986 Brussels, Belgium*
C. Gompel, 9th International Congress of Cytology, Brussels Conference Centre, Parc des Expos., 1020 Bruxelles, Belgium.

**Twenty-Seventh Congress of the European Society of Toxicology**
*27–29 May 1986 Harrogate, North Yorkshire, UK*
The Secretariat, Eurotox '86, Hazleton Laboratories Europe Ltd, Otley Rd, Harrogate, North Yorkshire, HG3 1PX, UK.

**E.O.R.T. Symposium of Gastrointestinal Tract Cancer – Update on Combined Modality Therapy**
*29–30 May 1986 Heidelberg, FRG*
Dr P. Schlag, Chirurgische Klinik, Universität Heidelberg, Im Neuenheimer Feld 110, 6900 Heidelberg, FRG.

**International Symposium on Hormonal Manipulation of Cancer**
*4–6 June 1986 Rotterdam, The Netherlands*
Congress Secretariat, Dr Daniel den Hoed Cancer Center, P.O. Box 5201, 3008 AE Rotterdam, The Netherlands.

**Fourth Annual ECP Symposium: Concepts and Theories in Carcinogenesis**
*12–13 June 1986 Bruges, Belgium*
ECP Secretariat, Avenue Lambeau 62, B-1200 Brussels, Belgium.

**Sixth International Congress on Immunology**
*6–11 July 1986 Toronto, Canada*
B. Cinader, Canadian Society for Immunology, Manitoba University, 730 William Avenue, Winnipeg, Manitoba RBE 0W3, Canada.

**Physics and Technology of Hyperthermia**
*26 July–9 August 1986 Urbino, Italy*
S.B. Field, MRC Cyclotron Unit, Hammersmith Hospital, Ducane Road, London W12, UK.

**International Conference on Theories of Carcinogenesis. Facts, Fashion or Fiction?**
*16–20 August 1986* *Oslo, Norway*
The Secretariat, The Norwegian Cancer Society, Huitfeldsgt. 49, 0253 Oslo 2, Norway

**Fourteenth International Cancer Congress**
*21–27 August 1986* *Budapest, Hungary*
Professor S. Eckhardt, National Institute of Oncology, Rath György u7/9, 1525 Budapest, Hungary.

**Fourth International Congress on Serology**
*1–4 September 1986* *Paris, France*
Dr J. Gest, Centre Rene Hiquenin, 35 rue Dailly, 92211, Saint-Cloud, France.

**Breast Diseases, IVth International Congress on Serology**
*1–4 September 1986* *Paris, France*
Dr J. Gest, B.P.41, 92215 Saint-Cloud Cedex, France.

**Non-mutagenic Carcinogens Conference**
*1–15 September 1986* *University of Surrey, Guildford, UK.*
Ms J. Williams, Robens Institute, University of Surrey, Guildford, GU2 5XH, Surrey, UK.

**2nd International Workshop on Multiple Endocrine Neoplasia, type 2**
*17–20 September 1986* *Cambridge, UK.*
Dr B.A.J. Ponder, Institute of Cancer Research, Clifton Avenue, Sutton, Surrey (tel 01-643 8901 ext 293).

**International Congress on Tumoral Markers**
*18–20 September 1986* *Naples, Italy*
OOMER Secretariat, ICIG, Hospital Paul-Brousse, 14–16 Avenue Paul-Vaillaut-Couturier, 94800 Villejuit, France. Tel: 46.77.00.00. ext: 306

**OOMER Second Annual Symposium on Cancer Epidemiology in Mediterranean Countries**
*20 September 1986* *Naples, Italy*
OOMER Secretariat, ICIG, Hospital Paul-Brousse, 14–16 Avenue Paul-Vaillaut-Couturier, 94800 Villejuif, France. Tel: 46.77.00.00. Ext: 306

**King's Fund Forum – Consensus and Controversies in Medicine; The Treatment of Primary Breast Cancer**
*1–3 October 1986* *R.I.B.A., London, UK*
Jackie Spiby, King's Fund College, 2 Palace Court, London W2 4HS, UK.

**Fifth N.C.I.-E.O.R.T.C. Symposium on New Drugs in Cancer Therapy**
*22–24 October 1986* *Free University of Amsterdam, The Netherlands*
Congress Office Free University, P.O. Box 7161, NL-1007 MC Amsterdam, The Netherlands, Tel: (20) 548 4656 or 548 5193, Telex: 11329

**IXth Meeting of the European Association for Cancer Research**
*1–3 June 1987* *Helsinki, Finland.*